# Author Correction: Plastid phylogenomics resolves ambiguous relationships within the orchid family and provides a solid timeframe for biogeography and macroevolution

**DOI:** 10.1038/s41598-021-93674-y

**Published:** 2021-07-06

**Authors:** Maria Alejandra Serna-Sánchez, Oscar A. Pérez-Escobar, Diego Bogarín, María Fernanda Torres-Jimenez, Astrid Catalina Alvarez-Yela, Juliana E. Arcila-Galvis, Climbie F. Hall, Fábio de Barros, Fábio Pinheiro, Steven Dodsworth, Mark W. Chase, Alexandre Antonelli, Tatiana Arias

**Affiliations:** 1 Laboratorio de Biología Comparativa, Corporación Para Investigaciones Biológicas (CIB), Cra. 72 A No. 78 B 141, Medellín, Colombia; 2 Biodiversity, Evolution and Conservation, EAFIT University, Cra. 49, No. 7 sur 50, Medellín, Colombia; 3 Royal Botanic Gardens Kew, London, TW9 3AE UK; 4 Jardín Botánico Lankester, Universidad de Costa Rica, P. O. Box 302-7050, Cartago, Costa Rica; 5 Endless Forms Group, Naturalis Biodiversity Center, P.O. Box 9517, 2300 RA Leiden, The Netherlands; 6 Gothenburg Global Biodiversity Centre, Department of Biological and Environmental Sciences, University of Gothenburg, 405 30 Gothenburg, Sweden; 7 Centro de Bioinformática y Biología Computacional (BIOS), Ecoparque Los Yarumos Edificio BIOS, Manizales, Colombia; 8 Instituto de Botânica, Núcleo de Pesquisa Orquídario Do Estado, Postal 68041, São Paulo, SP 04045-972 Brasil; 9 Instituto de Biologia, Departamento de Biologia Vegetal, Universidade Estadual de Campinas, Campinas, SP 13083-862 Brazil; 10 School of Life Sciences, University of Bedfordshire, University Square, Luton, LU1 3JU UK; 11 Department of Plant Sciences, University of Oxford, South Parks Road, Oxford, OX1 3RB UK; 12 Tecnológico de Antioquia, Calle 78B NO. 72A - 220, Medellín, Colombia

Correction to: *Scientific Reports* https://doi.org/10.1038/s41598-021-83664-5, published online 25 March 2021

The original version of this Article contained an error in Figure 1 where the image showing "Paphiopedilum villosum" was incorrectly labelled as "Apostasia shenzhenica".

The original Figure 1 and accompanying legend appear below.

The original Article has been corrected.

**Figure 1:**
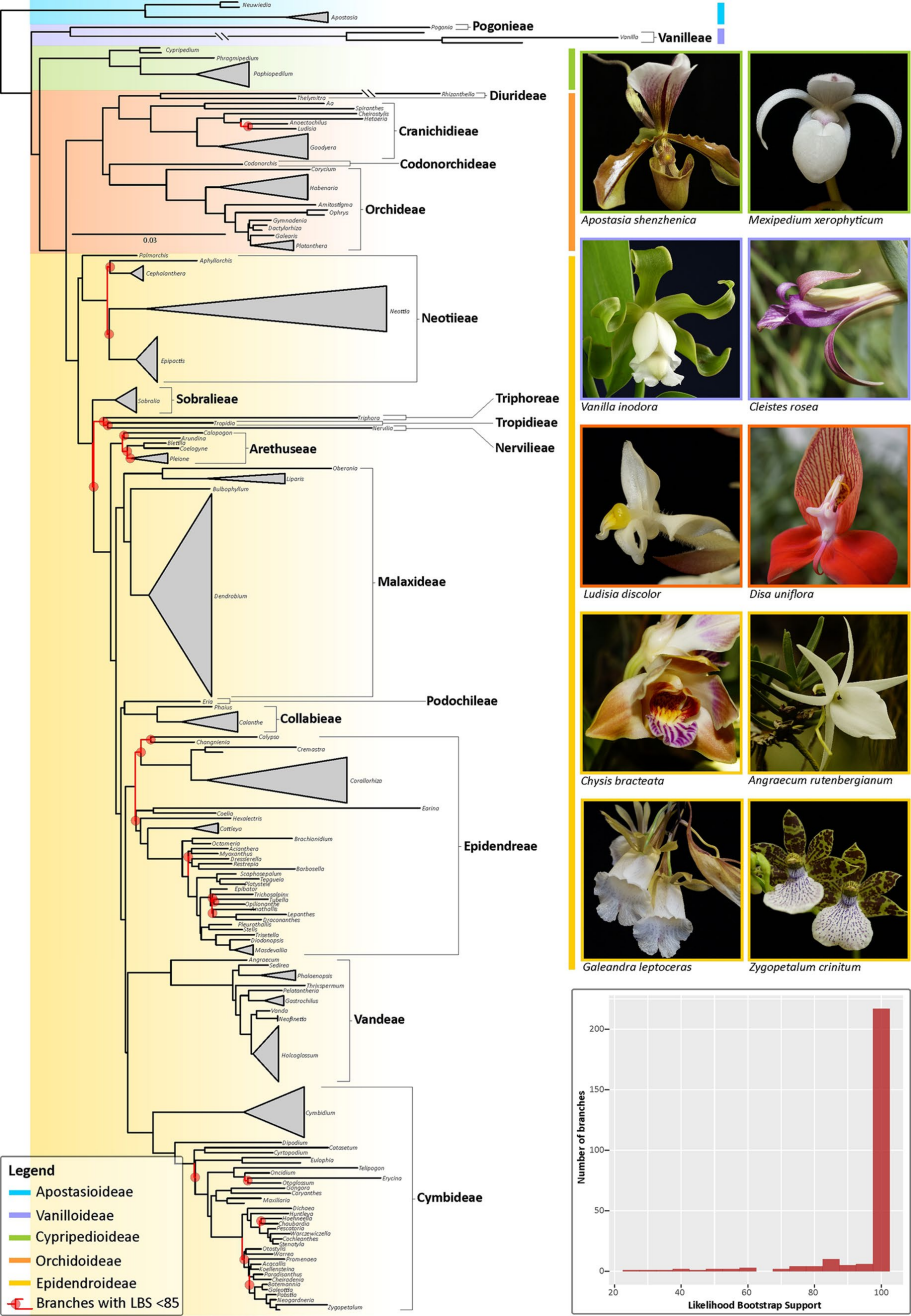
Maximum Likelihood phylogeny of the orchid family inferred from 78 coding plastid genes. Likelihood bootstrap support values (LBS) <85% at nodes are highlighted in red together with their corresponding subtending branches. Orchid genera, tribes and subfamilies are indicated in the phylogeny together with photographs of selected representative species per subfamily. (Inset): Bar plot showing the frequency of LBS values at branches as computed by bin intervals of 5 units. Photos: O. Pérez-Escobar & D. Bogarín.

