# Supplementary material for: Plastid phylogenomics resolves ambiguous relationships within the orchid family and provides a solid timeframe for biogeography and macroevolution
Source: Sci Rep. 2021 Mar 25;11:6858. doi: 10.1038/s41598-021-83664-5 (PMC7994851; doi:10.1038/s41598-021-83664-5)
Supplement: Supplementary file 1 — Supplementary caption 1. [file 41598_2021_83664_MOESM1_ESM.pdf]

Plastid phylogenomics resolves ambiguous relationships within the orchid family and provides a solid timeframe for biogeography and macroevolution

Maria Alejandra Serna-Sánchez[1,2+], Oscar A. Pérez-Escobar[3*+], Diego Bogarín[4,5+], María Fernanda Torres[6], Astrid Catalina Alvarez-Yela[7], Juliana E. Arcila[1], Climbie F. Hall[8], Fabio Pinheiro[9], Steven Dodsworth[10], Mark W. Chase[3], Alexandre Antonelli[3,6,11], Tatiana Arias[1,7,12*]

[1] Laboratorio de Biología Comparativa. Corporación para Investigaciones Biológicas (CIB), Cra. 72 A No. 78 B 141, Medellín, Colombia.
[2] Biodiversity, Evolution and Conservation. EAFIT University, Cra. 49, No. 7 sur 50, Medellín, Colombia.
[3] Royal Botanic Gardens, Kew, TW9 3AE, London, UK.
[4] Jardín Botánico Lankester, Universidad de Costa Rica, P. O. Box 302-7050, Cartago, Costa Rica.
[5] Naturalis Biodiversity Center, Endless Forms group, P.O. Box 9517, 2300 RA Leiden, The Netherlands; Herbario UCH, Universidad Autónoma de Chiriquí, David, Panamá.
[6] Gothenburg Global Biodiversity Centre, Department of Biological and Environmental Sciences, University of Gothenburg, 405 30 Gothenburg, Sweden.
[7] Centro de Bioinformática y Biología Computacional (BIOS). Ecoparque Los Yarumos Edificio BIOS, Manizales, Colombia.
[8] Universidade Federal de Mato Grosso do Sul, Tres Lagos, Mato Groso, Brazil.
[9] Universidade Estadual de Campinas, Instituto de Biologia, Departamento de Biologia Vegetal, 13083-862, Campinas-SP, Brazil
[10] School of Life Sciences, University of Bedfordshire, University Square, Luton, LU1 3JU, UK.
[11] Department of Plant Sciences, University of Oxford, South Parks Road, OX1 3RB Oxford, United Kingdom
[12] Tecnológico de Antioquia, Calle 78B NO. 72A - 220 Medellín- Colombia
[*] Corresponding Authors: T.A. (tatiana.arias48@tdea.edu.co) & O.A.P.E. (o.perez-escobar@kew.org)
[+] These authors contributed equally to the study.

ONLINE SUPPLEMENTARY MATERIAL:

Including Supplementary Tables S1-S5
Including Supplementary Figures S1-S4

Supplementary tables

Table S1. Absolute age estimations derived from a strict clock and a Birth-Death tree model applied to a dataset of 264 terminals and 78-coding-sequence plastid gene concatenated alignment. The table is composed of 16 columns and 529 rows and is freely available at https://doi.org/10.6084/m9.figshare.14068892.

Table S2. Absolute age estimations derived from a relaxed clock and a Birth-Death tree model applied to a dataset of 264 terminals and 78-coding-sequence plastid gene concatenated alignment. The table is composed of 16 columns and 529 rows and is freely available at https://doi.org/10.6084/m9.figshare.14068892.

Table S3. Per-site maximum and minimum phylogenetic informativeness (PI) values per locus, estimated from a dataset of 264 terminals and 78-coding-sequence plastid gene alignments. The table is composed of 80 columns and five rows and is freely available at https://doi.org/10.6084/m9.figshare.14068892.

Table S4. Net maximum and minimum phylogenetic informativeness (PI) values per locus, estimated from a dataset of 264 terminals and 78-coding-sequence plastid gene alignments. The table is composed of 80 columns and five rows and is freely available at https://doi.org/10.6084/m9.figshare.14068892.

Table S5. Species names and voucher information for material sampled in this study. Newly sequenced taxa are highlighted in light blue. The table is composed of 266 columns and seven rows and is freely available at https://doi.org/10.6084/m9.figshare.14068892.

Supplementary figures

Figure S1. Detailed maximum likelihood tree of the orchid family inferred from 78 plastid genes. LBP <100 are shown at nodes, with LBP <85 highlighted in red together with their corresponding subtending branches.

Figure S2. Chronogram of the orchid family as inferred from a strict molecular clock and a birth-death model. LBP at nodes <85 are highlighted in red together with their corresponding subtending branches. Blue bars at nodes denote 95% high density probability (HDP) absolute age intervals.

Figure S3. Chronogram of the orchid family as inferred from a relaxed molecular clock and a birth-death model. LBP at nodes <85 are highlighted in red together with their corresponding subtending branches. Blue bars at nodes denote 95% high density probability (HDP) absolute age intervals.

Figure S4. Phylogenetic informativeness (PI) of 78 plastid gene alignments used in this study to infer orchid relationships. A) Chronogram of Orchidaceae as inferred by PATH8 from the ML tree derived from RAxML; B) Per-site PI; C) Net PI.

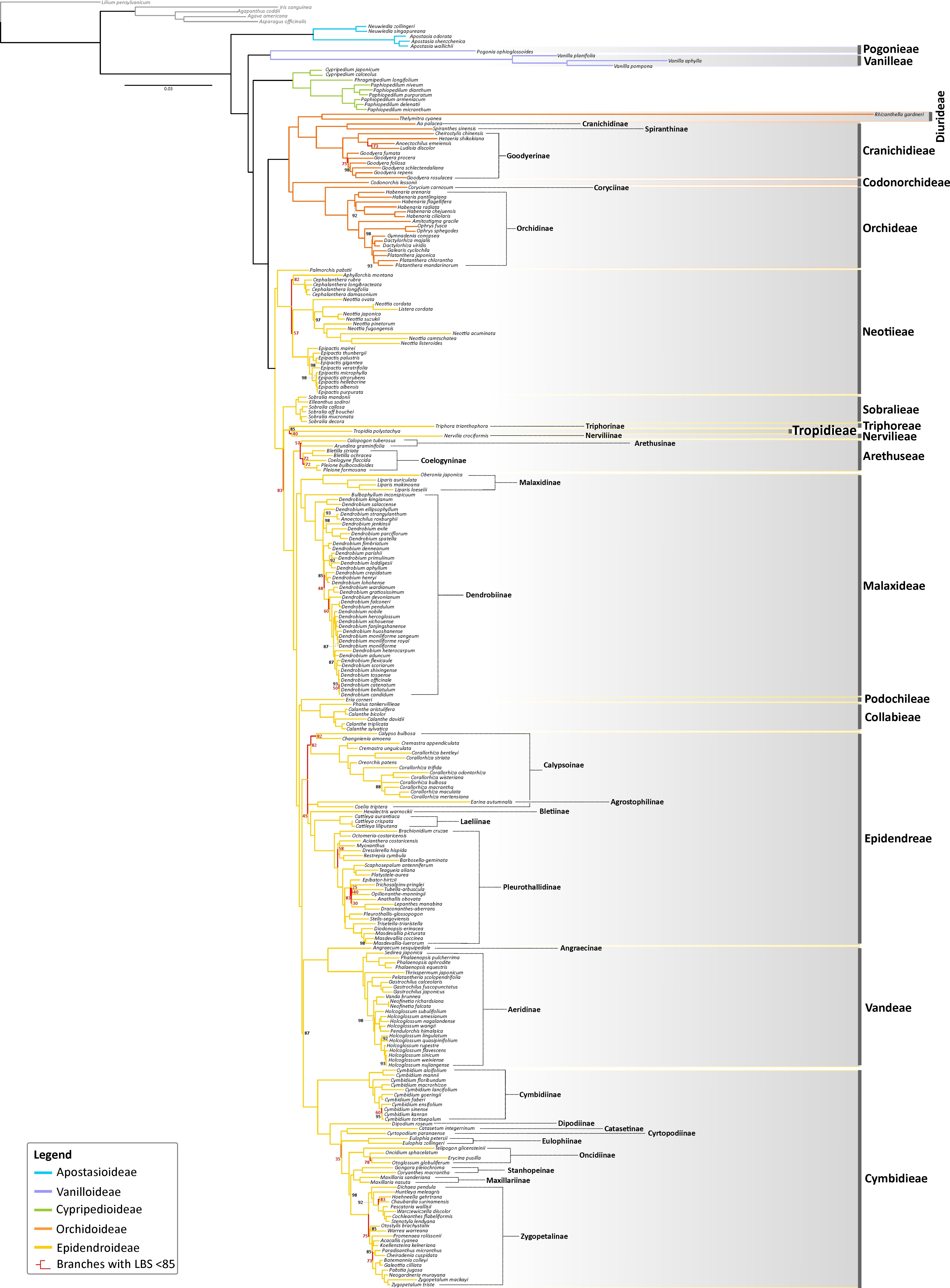

Pogonieae
Vanilleae
Diurideae
Cranichidinae
Spiranthinae
Goodyerinae
Cranichidieae
Codonorchideae
Coryciinae
Orchidinae
Orchideae
Neotiieae
Sobralieae
Triphorinae
Triphoreae
Tropidieae
Nerviliinae
Nervilieae
Arethusinae
Coelogyninae
Arethuseae
Malaxidinae
Dendrobiinae
Malaxideae
Podochileae
Collabieae
Calypsoinae
Agrostophilinae
Bletiinae
Laeliinae
Epidendreae
Pleurothallidinae
Angraecinae
Aeridinae
Vandeae
Cymbidiinae
Dipodiinae
Catasetinae
Cyrtopodiinae
Eulophiinae
Oncidiinae
Stanhopeinae
Maxillariinae
Zygopetalinae
Cymbidieae
0.03
Legend
Apostasioideae
Vanilloideae
Cypripedioideae
Orchidoideae
Epidendroideae
Branches with LBS <85

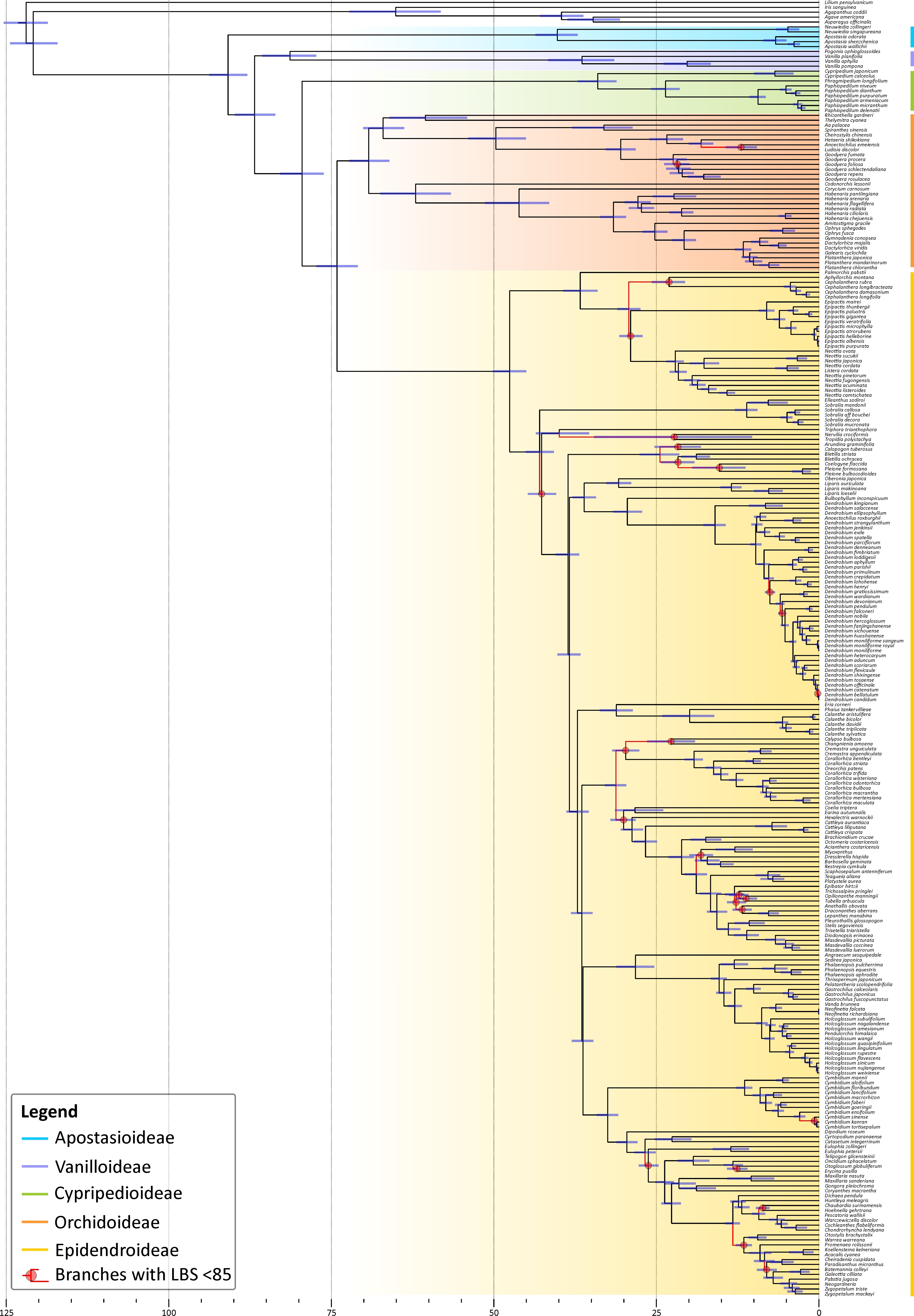

Lilium pensylvanicum
Iris sanguinea
Agapanthus coddii
Agave americana
Asparagus officinalis
Neuwiedia zollingeri
Neuwiedia singapureana
Apostasia odorata
Apostasia shenzhenica
Apostasia wallichii
Pogonia ophioglossoides
Vanilla planifolia
Vanilla aphylla
Vanilla pompona
Cypripedium japonicum
Cypripedium calceolus
Phragmipedium longifolium
Paphiopedilum niveum
Paphiopedilum dianthum
Paphiopedilum purpuratum
Paphiopedilum armeniacum
Paphiopedilum micranthum
Paphiopedilum delenatii
Rhizanthella gardneri
Thelymitra cyanea
Aa palacea
Spiranthes sinensis
Cheirostylis chinensis
Hetaeria shikokiana
Anoectochilus emeiensis
Ludisia discolor
Goodyera fumata
Goodyera procera
Goodyera foliosa
Goodyera schlectendaliana
Goodyera repens
Goodyera rosulacea
Codonorchis lessonii
Corycium carnosum
Habenaria pantlingiana
Habenaria arenaria
Habenaria flagellifera
Habenaria radiata
Habenaria ciliolaris
Habenaria chejuensis
Amitostigma gracile
Ophrys sphegodes
Ophrys fusca
Gymnadenia conopsea
Dactylorhiza majalis
Dactylorhiza viridis
Galearis cyclochila
Platanthera japonica
Platanthera mandarinorum
Platanthera chlorantha
Palmorchis pabstii
Aphyllorchis montana
Cephalanthera rubra
Cephalanthera longibracteata
Cephalanthera damasonium
Cephalanthera longifolia
Epipactis mairei
Epipactis thunbergii
Epipactis palustris
Epipactis gigantea
Epipactis veratrifolia
Epipactis microphylla
Epipactis atrorubens
Epipactis helleborine
Epipactis albensis
Epipactis purpurata
Neottia ovata
Neottia suzukii
Neottia japonica
Neottia cordata
Listera cordata
Neottia pinetorum
Neottia fugongensis
Neottia acuminata
Neottia listeroides
Neottia camtschatea
Elleanthus sodiroi
Sobralia mandonii
Sobralia aff bouchei
Sobralia callosa
Sobralia decora
Sobralia mucronata
Triphora trianthophora
Nervilia crociformis
Tropidia polystachya
Arundina graminifolia
Calopogon tuberosus
Bletilla striata
Bletilla ochracea
Coelogyne flaccida
Pleione formosana
Pleione bulbocodioides
Oberonia japonica
Liparis auriculata
Liparis makinoana
Liparis loeselii
Bulbophyllum inconspicuum
Dendrobium kingianum
Dendrobium salaccense
Dendrobium ellipsophyllum
Anoectochilus roxburghii
Dendrobium strongylanthum
Dendrobium jenkinsii
Dendrobium exile
Dendrobium spatella
Dendrobium parciflorum
Dendrobium denneanum
Dendrobium fimbriatum
Dendrobium loddigesii
Dendrobium aphyllum
Dendrobium parishii
Dendrobium primulinum
Dendrobium crepidatum
Dendrobium lohohense
Dendrobium henryi
Dendrobium gratiosissimum
Dendrobium wardianum
Dendrobium devonianum
Dendrobium pendulum
Dendrobium falconeri
Dendrobium nobile
Dendrobium hercoglossum
Dendrobium fanjingshanense
Dendrobium xichouense
Dendrobium huoshanense
Dendrobium moniliforme sangeum
Dendrobium moniliforme royal
Dendrobium moniliforme
Dendrobium heterocarpum
Dendrobium aduncum
Dendrobium scoriarum
Dendrobium flexicaule
Dendrobium shixingense
Dendrobium tosaense
Dendrobium officinale
Dendrobium catenatum
Dendrobium bellatulum
Dendrobium candidum
Eria corneri
Phaius tankervilleae
Calanthe aristulifera
Calanthe bicolor
Calanthe davidii
Calanthe triplicata
Calanthe sylvatica
Calypso bulbosa
Changnienia amoena
Cremastra unguiculata
Cremastra appendiculata
Corallorhiza bentleyi
Corallorhiza striata
Oreorchis patens
Corallorhiza trifida
Corallorhiza wisteriana
Corallorhiza odontorhiza
Corallorhiza bulbosa
Corallorhiza macrantha
Corallorhiza mertensiana
Corallorhiza maculata
Coelia triptera
Earina autumnalis
Hexalectris warnockii
Cattleya aurantiaca
Cattleya liliputana
Cattleya crispata
Brachionidium cruzae
Octomeria costaricensis
Acianthera costaricensis
Myoxanthus
Dresslerella hispida
Barbosella geminata
Restrepia cymbula
Scaphosepalum antenniferum
Teagueia aliana
Platystele aurea
Epibator hirtzii
Trichosalpinx pringlei
Opilionanthe manningii
Tubella arbuscula
Anathallis obovata
Draconanthes aberrans
Lepanthes manabina
Pleurothallis glossopogon
Stelis segoviensis
Trisetella triaristella
Diodonopsis erinacea
Masdevallia picturata
Masdevallia coccinea
Masdevallia luerorum
Angraecum sesquipedale
Sedirea japonica
Phalaenopsis pulcherrima
Phalaenopsis equestris
Phalaenopsis aphrodite
Thrixspermum japonicum
Pelatantheria scolopendrifolia
Gastrochilus calceolaris
Gastrochilus japonicus
Gastrochilus fuscopunctatus
Vanda brunnea
Neofinetia falcata
Neofinetia richardsiana
Holcoglossum subulifolium
Holcoglossum nagalandense
Holcoglossum amesianum
Pendulorchis himalaica
Holcoglossum wangii
Holcoglossum quasipinifolium
Holcoglossum lingulatum
Holcoglossum rupestre
Holcoglossum flavescens
Holcoglossum sinicum
Holcoglossum nujiangense
Holcoglossum weixiense
Cymbidium mannii
Cymbidium aloifolium
Cymbidium floribundum
Cymbidium lancifolium
Cymbidium macrorhizon
Cymbidium faberi
Cymbidium goeringii
Cymbidium ensifolium
Cymbidium sinense
Cymbidium kanran
Cymbidium tortisepalum
Dipodium roseum
Cyrtopodium paranaense
Catasetum integerrimum
Eulophia zollingeri
Eulophia petersii
Telipogon glicensteinii
Oncidium sphacelatum
Otoglossum globuliferum
Erycina pusilla
Maxillaria nasuta
Maxillaria sanderiana
Gongora pleiochroma
Coryanthes macrantha
Dichaea pendula
Huntleya meleagris
Chaubardia surinamensis
Hoehnella gehrtrana
Pescatoria wallisii
Warczewiczella discolor
Cochleanthes flabelliformis
Chondrorhyncha lendyana
Otostylis brachystalix
Warrea warreana
Promenaea rollissonii
Koellensteina kelneriana
Acacallis cyanea
Cheiradenia cuspidata
Paradisanthus micranthus
Batemannia colleyi
Galeottia ciliata
Pabstia jugosa
Neogardneria
Zygopetalum triste
Zygopetalum mackayi
Legend
Apostasioideae
Vanilloideae
Cypripedioideae
Orchidoideae
Epidendroideae
Branches with LBS <85
125
100
75
50
25
0

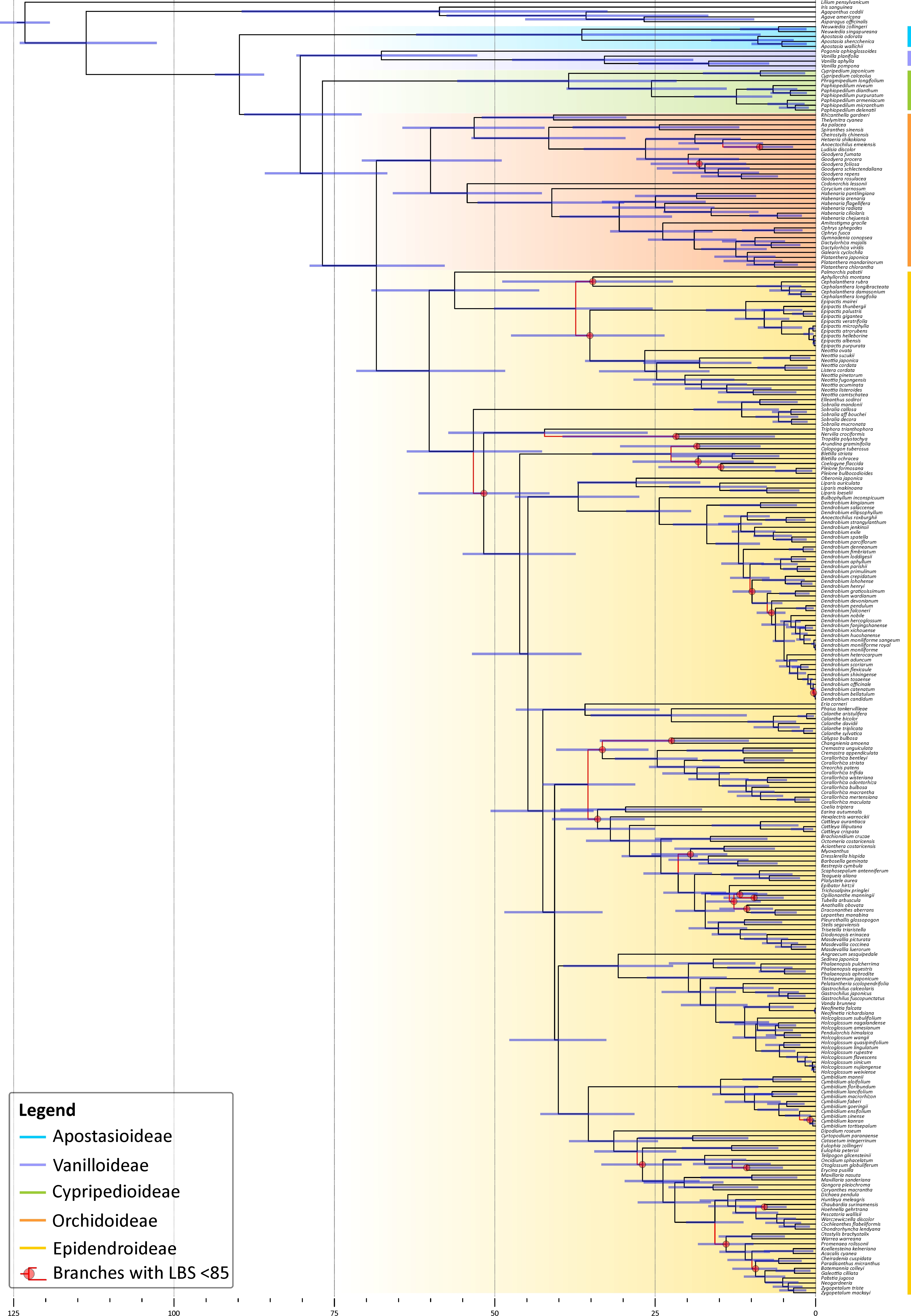
Legend
Apostasioideae
Vanilloideae
Cypripedioideae
Orchidoideae
Epidendroideae
Branches with LBS <85
125
100
75
50
25
0

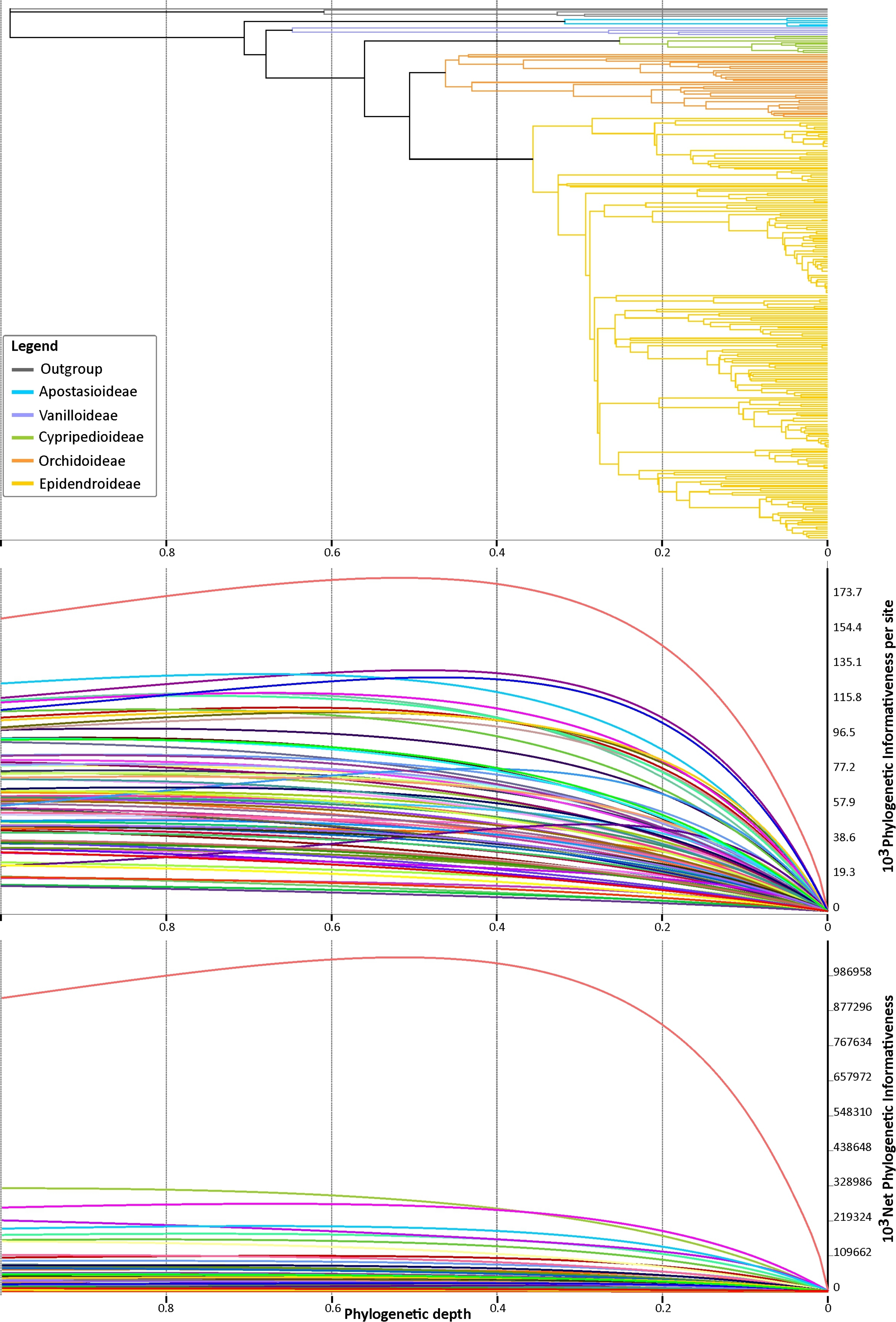

Legend
Outgroup
Apostasioideae
Vanilloideae
Cypripedioideae
Orchidoideae
Epidendroideae
0.8
0.6
0.4
0.2
0
173.7
154.4
135.1
115.8
96.5
77.2
57.9
38.6
19.3
0
10^3 Phylogenetic Informativeness per site
986958
877296
767634
657972
548310
438648
328986
219324
109662
0
10^3 Net Phylogenetic Informativeness
Phylogenetic depth
